# Insight into the Physical Properties of Fluoro-Perovskites Compounds of Tl-Based TlMF_3_ (M = Au, Ga) Compounds Studied for Energy Generation Utilizing the TB-MBJ Potential Approximation Approach

**DOI:** 10.3390/ma16020686

**Published:** 2023-01-10

**Authors:** Hukam Khan, Mohammad Sohail, Muhammad Shoaib Arif, Kamaleldin Abodayeh

**Affiliations:** 1 Department of Physics, University of Lakki Marwat, Lakki Marwat 28420, Khyber Pakhtunkhwa, Pakistan; 2 Department of Mathematics and Sciences, College of Humanities and Sciences, Prince Sultan University, Riyadh 11586, Saudi Arabia; 3 Department of Mathematics, Air University, PAF Complex E-9, Islamabad 44000, Pakistan

**Keywords:** density functional theory, fluoro-pervoskite, optical characteristic, elastic properties, electronic properties

## Abstract

Fluoro-perovskites compounds based on the Tl element TlMF_3_ (M = Au, Ga) were examined computationally, and their different aspects, studied utilizing TB-mBJ potential approximations, can be used for the generation of energy because of their ever-increasing power conversion efficiency. Birch Murnaghan’s graph and tolerance factor show that these composites are structurally cubic and stable. The optimum volume of the compounds corresponding to the optimum energies and the optimized lattice constants were computed. The algorithm IRelast was used to predict the elastic information, and these results demonstrated that the presented compounds are stable mechanically and show anisotropic and ductile properties. TlAuF_3_ and TlGaF_3_ have an indirect band energy gap at (M-X) positions, with a forbidden energy gap of −0.55 eV for TlAuF_3_ and 0.46 eV for TlGaF_3_. The compounds show a metallic nature due to a small indirect band gap. Different component states corresponding to the upper and lower bands of the Fermi energy level are influenced by the total density in the different states and the density in various directions (TDOS & PDOS). These composites exhibit strong absorption, conductivity, and reflective coefficients at higher energy series together with a low refractive index, given by an inquiry into optical properties. The applications of these composites are thought to be good for conduction purposes in industries due to the indirect band gap. For the first time, computational analysis of these novel compounds offers a thorough understanding of their many characteristics.

## 1. Introduction

Fluoro-perovskites compounds are famous due to their standard chemical structure ABF3, where the fluorine atom is an anion with a negative sign and A and B are cations (positive charge ions) in the molecule ABF3. Having a mechanically stable crystal structure and outstanding optoelectronic characteristics ranging from semiconducting (1–4 eV) to non-conducting materials (over 4 eV), fluoro-perovskites materials are a fascinating family of materials. Due to their practical significance as lenses in the field of photolithography, light measuring devices (photo dosimeters), flashlight materials, and in semiconductor fabrication, fluoro-perovskites have attracted great interest recently [1,2,3]. Fluoro-perovskites compounds have undergone substantial experimental study and have many computational uses, as seen in [4].

Fluoro-perovskites’ energy band gap is frequently wide [5,6,7]. Due to their wide energy gaps, these compounds are significant from a technical standpoint. As vacuum UV materials for optical lenses, KMgF3 and BaLiF3 are used in optical lithography steppers [7,8,9]. When a group of lanthanides, like Ce and Er ions, is doped, KMgF3 also exhibits a radiation dosimeter and sparkling ability [10]. Murtaza et al. [11] conducted a theoretical examination of the compound based on the Ag fluoro-perovskites MgAgF3 and ZnAgF3. The suggested materials fulfill various needs in modern technology due to their large energy absorption spectrum. After examining their opto-electronic characteristics, fluoro-perovskites based on Sn were learned to be insulators, and it was predicted of Auger-Free luminescence [12]. F. Hamioud et al. studied the optoelectronics, fundamental structural, and magnetic characteristics of TlMnX3 (X = F and Cl) in [13,14]. They also predicted optical spectra-based applications for optics technology. Numerous studies have demonstrated that the compound based on thallium is being produced for light detection [15,16]. Due to the presence of a thallium atom, these compounds have a larger effective atomic number, enhancing detection effectiveness. Additionally, these composites are technologically attractive candidates due to their single progress requisite and straightforward cubic structure.

The TB-mBJ approach, which offers insight into different physical properties like structural, elastic, electrical, and optical properties, is used to study TlMF_3_ (M = Ga, Au) compounds in detail. We have found that TlAuF_3_ is an excellent choice for electrical needs because of its good electrical conduction with high transparency throughout a constrained range of energies, according to the computer simulation program WIEN2K. Despite the interest in Tl-based fluoro-perovskites for several applications, to the best of our information, no works can be found in the literature on the subject. This work is made up of four primary parts. The calculation procedure is covered in Section 2, the results and analysis in Section 3, and the study’s conclusions are presented in Section 4.

## 2. Methodology

**Figure 1:**
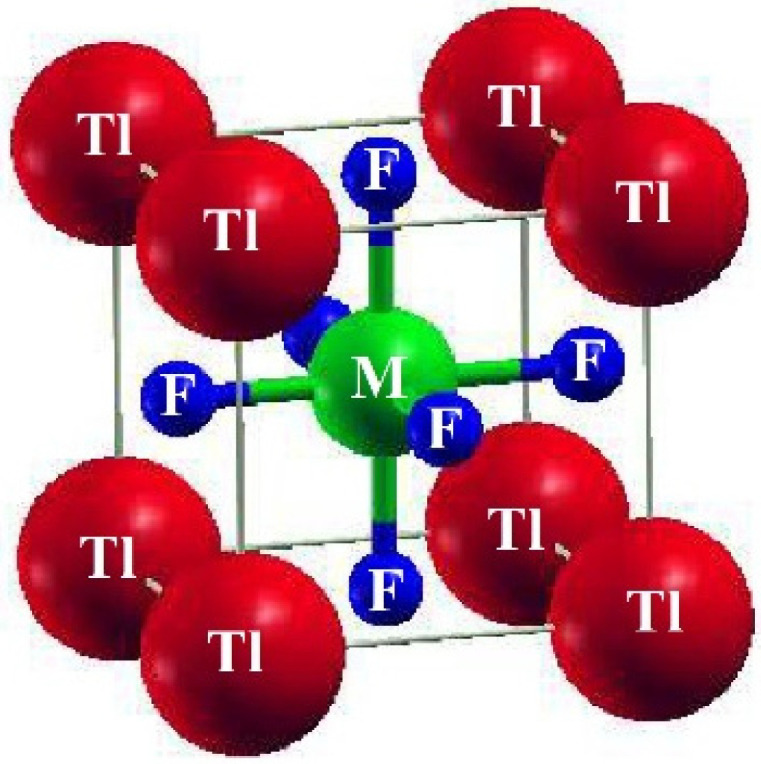
The structure of the basic unit cell of TlMF_3_ (M= Au, Ga) fluoro-perovskites.

**Figure 2:**
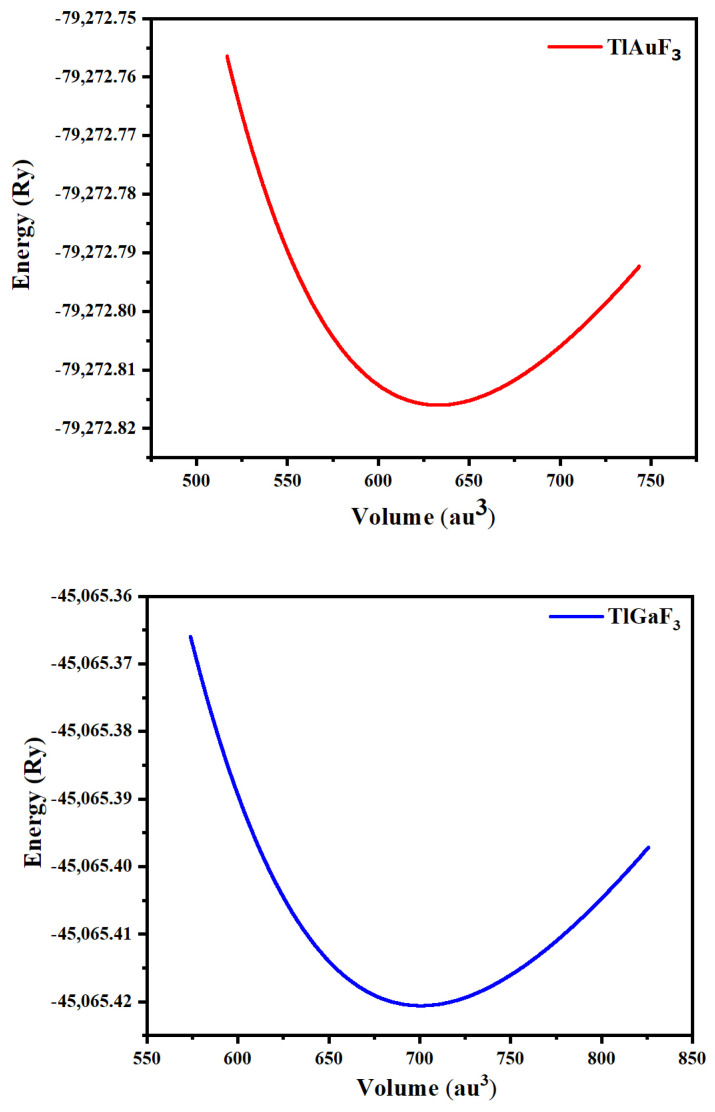
Representing TlMF_3_ (M = Au, Ga) dependence of the optimized energy against volume.

A cubic shape of perovskites having a space group of Pm-3m (#221) characterizes ternary fluoro-perovskite TlMF_3_ (M = Au, Ga) compounds. The basic unit of the material made up of one atom of Tl is located at (0, 0, 0) and the atom M (M = Au, Ga) at (1/2, 1/2, 1/2), Wyckoff coordinates, respectively. In contrast, at (1/2, 0, 1/2), (0, 1/2, 1/2), and (1/2, 1/2, 0), the three fluorine atoms are located in the compound (Figure 1) as depicted. The common structure of density functional theory is applied for computational studies to examine structural, elastic, electrical, and optical characteristics. In this study, the WIEN2K code and the FP-LAPW method were utilized to the fullest extent possible. [17]. The graph demonstrating energy vs. volume is illustrated in Figure 2 for both compounds using Murnaghan’s equation of states, thereby determining the structural properties of the said compounds [18].

The right value of RMT was chosen for this inquiry to ensure that no charges escaped from the whole and core energy. RMT values for Tl, Au, and Ga were 2.02, 2.50, and 2.52, respectively, while RMT values for F in TlAuF_3_ and TlGaF_3_ were 2.02 and 2.09, respectively. Due to the muffin tin sphere’s 1500 K-points and 13.0 Gmax, the waveform was overextended in harmonics up to lmax = 10. The energy difference between the valence and core bands was 6.0 Ry.

## 3. Results and Discussions

### 3.1. Electronic Properties

**Figure 3:**
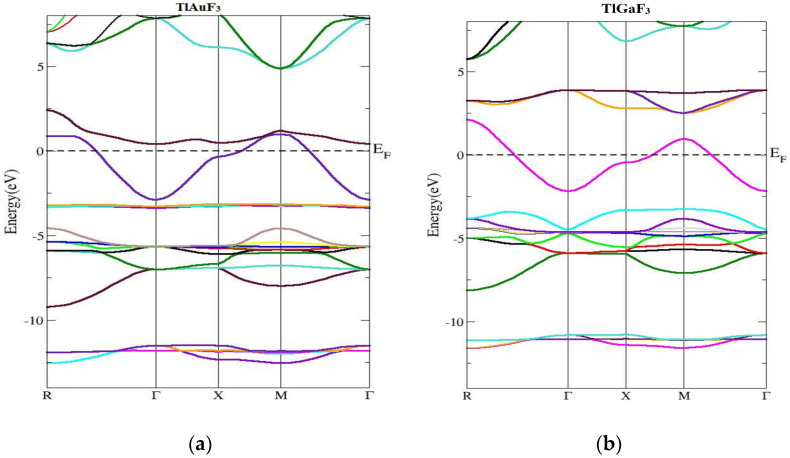
Computed band structures of TlMF_3_ (M= Au, Ga). (**a**) Represent band structure of TlAuF_3_ (**b**) Represent band structure of TlGaF_3_.

The band of energy constructions and density of states of the ccompounds under examination are reported in this section. Figure 3 shows the computed band’s structure for TlMF_3_ (M = Au, Ga) at stable geometry in the first Brillion zone and the symmetry directions. It was found that there is no chance of zero. Preferably, at the maxima of the VB, energy should be at Fermi level or close. The compound band gap for TlAuF_3_ and TlGaF_3_ had computed values of −0.55 eV and 0.46 eV, respectively, obtained by applying the TB-MBJ approximation potential technique. In both cases, the maxima of the lower band touch across the EF, and demonstrate that both compounds have the nature of metal.

The structure of the energy bands in Figure 2a demonstrates that the lowest of the upper bands of TlAuF_3_ occurs at the point Г of the symmetry and the highest state of the VB occurs at M symmetry, which indicates an indirect band. From Figure 2, we can conclude that the TlAuF_3_ compound has a metallic nature. The conduction band minima of TlGaF_3_ are found at the point of symmetry M, while the maxima of the lower band occurs at R of the Brillion zone, proving that the compound has an indirect gap.

This section concerned the energy band constructions of the composites. The TlMF_3_ (M = Au, Ga) calculated band structure and symmetry directions at stable geometry along the first Brillion zone are given in Figure 3.

### 3.2. Density of States

**Figure 4:**
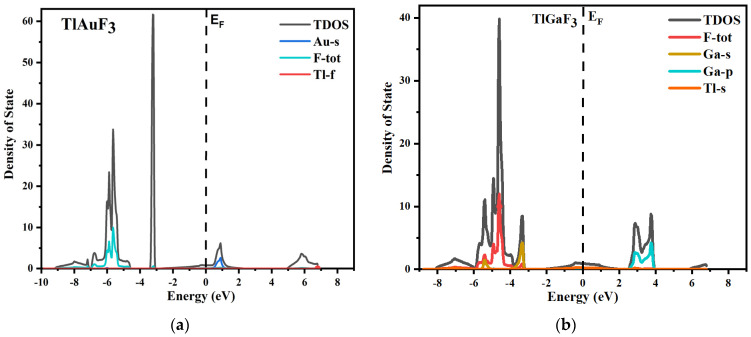
(**a**) Total and partial density of states of TlAuF_3_, (**b**) Total and partial density of states of TlAuF_3_.

The total and partial density of states for both source compounds under inquiry is shown in Figure 4, which displays the parts of the electronic positions relative to the VB and CB bands. The vertical dotted line shows the Fermi energy level, shown as EF. The region to the left of the Fermi levels displays the VB, while the conduction band is to the right of the Fermi energy level.

The various states of a compound’s component can be used to understand it better. The plot of the total and partial DOS for TlAuF_3_ shows that the F-total is due to the involvement of all the states and dominants in the valence band. However, the F state does not significantly contribute and the Ga-s provides only weak support. The Ga element plays a major part in CB. When it comes to the TlGaF_3_, the VB is primarily in a state of gap, with very little help from the Ga-s state.

### 3.3. Elastic Properties

The crucial elastic factors Cij can be utilized to explain a material’s elastic properties. Elastic constants play a key role in predicting how a chemical will respond to macroscopic stress. The elastic parameters can be used to explain how a compound deforms when stressed. Once the external force has subsided, it returns to its initial shape and undergoes further deformation [19]. The structural stability, atomic sphere bonding characteristics, and isotropic or anisotropic features can all be explained using elastic constants. The three different elastic numbers are C11, C12, and C44 for a cubic system. The unit cell is tilted with a suitable strain tensor to generate an energy strain relation, which is used to determine Cij. The IRelast-package [20], developed by Jamal Murtaza, was used in the present work and was executed within the WIEN2k code. Tl-based fluoro-perovskite had the perfect cubic crystal shape, as depicted in Figure 1. The total energy vs. volume graph deviation is seen in Figure 2. The equilibrium lattice parameters were obtained by fitting the Birch-Murnaghan rocking. All these numbers are presented in Table 1.

The elastic constant calculates a crystal’s reaction to applied forces and can tell us a lot about a material’s mechanical properties. Calculations are made utilizing C11, C12, and C44 to determine the stiffness and stability of cubic symmetry crystals and their elastic characteristics. The estimated values for the elastic parameters are depicted in Table 2. The results obtained from the experiment and the estimated elastic constants for TlAuF_3_ were compared. According to [21,22], the values of the elastic constant for TlGaF_3_ are 85.28 GPa, 24.27 GPa, and 0.29 GPa, while those for TlAuF_3_ are 86.88 GPa, 30.29 GPa, and −1.70 GPa. In the case of TlAuF_3_, the TB-MBJ method produces additional exact elastic characteristics that are more in line with experimental findings than the GGA-WC method. The B bulk modulus can be calculated from the elastic factors using the relations for ECs. Every EC is favorable and complies with the requirements. C11 > 0, C44 > 0, (C11 − C12) > 0, (C11 + 2C12) > 0, and C12 > B > C11 are all positive values for elastic stability [23]. Table 2 illustrates the output for Young’s modulus (E), anisotropy factor (A), Poisson’s ratio (v), and Pugh’s index ratio (B/G) using the following equations [24]:

(1) $$B=\frac{C_{11}+2C_{12}}{3}$$

(2) $$A=\frac{2C_{44}}{C_{11}-C_{12}}$$

(3) $$v=\frac{3B-2G}{2\left(2B+G\right)}$$

(4) $$v=\frac{3B-2G}{2\left(2B+G\right)}$$

(5) $$G_{v}=\frac{C_{11}-C_{12}+3C_{44}}{5}$$

(6) $$G_{R}=\frac{5C_{44}\left(C_{11}-C_{12}\right)}{4C_{44}+3C_{11}-C_{12}}$$

(7) $$G=\frac{G_{v}+G_{R}}{2}$$

The bulk modulus B implies a fractured opposition, whereas the shear modulus G denotes an opposition to plastic deformation. The B/G ratio indicates material status, whether it is brittle or ductile [25]. The materials will show brittle characteristics only if B/G is less than 1.750, and will be ductile if greater than 1.750. The Pugh’s criteria value of B/G is greater than 1.750, demonstrating the ductility of both materials. A material’s brittleness is concluded by the v [26]. If v is larger than 0.260, the material is ductile and brittle, as illustrated in Table 2. The ductility of each compound was double-checked. A stands for the elastic anisotropy factor, A = 1 indicating an isotropic material, and anisotropy being any number other than one. The amount of departure from 1 determines the crystal’s elastic anisotropy degree. We demonstrated that both of our compounds have a value of A that is different from one, proving that both have anisotropy. Young’s modulus is the most accurate measure of a material’s stiffness. Both compounds are the stiffest when the value is high for a certain substance, which causes the material to stiffen.

**Table 2:** Computer-generated mechanical parameters of TlMF_3_ (M = Au, Ga), using IRelast package.

Compounds	C11	C12	C44	B	G	E	A	v	B/G
TlAuF_3_	86.88	30.29	1.70	172.03	3.68	30.30	−0.06	0.47	46.78
TlGaF_3_	85.28	24.27	0.29	172.03	6.43	36.30	0.01	0.47	26.74

Poisson’s ratio provides the best information to determine the power of bonding uniqueness than any other elastic feature. For covalent bonding, the Poisson ratio is v = 0.1, but for ionic materials, it is usually v = 0.25. Table 2 reveals that the value of v for both compounds is more than 0.25, indicating that all of our materials have an ionic bonding characteristic.

### 3.4. Optical Properties

The TB-mBJ potential method is used to compute the visual properties of solid materials. Solids’ electronic complex dielectric function ε(ω) can be used to describe their visual properties. The communication of this complex function relies on both intra-band and inter-band movements. The two main types of inter-band contributions of metals are direct and indirect transitions [27]. Because they have little effect on the properties of ε(ω) and scattering of phonons, we discounted indirect inter-band movement. An appropriate representation of the linear response of the electronic system to the applied external field can be found in the anisotropic properties of this dielectric compound, which are simply a complex version of a symmetrical tensor of the 2nd order. The Ehrenreich and Cohen equation defines the complex dielectric function, which can be used to characterize a compound’s optical properties [18]:

(8) $$\varepsilon \left(\omega \right)=\varepsilon_{1}\left(\omega \right)+i\varepsilon_{2}\left(\omega \right)$$

Real and imaginary components are represented by the letters ε_1_(ω) and ε_2_(ω), respectively. The imaginary part of the dielectric function can be used to compute the real portion, which is dependent on ω′^2^–ω^2^ and provides the integral denominator. It also has a direct relation to Eg. Using the Kramers-Kronig equation, which is as follows, one can calculate the real part, ε_1_(ω).

(9) $$\varepsilon_{1}\left(\omega \right)=1+\frac{2}{\pi }P\int_{0}^{\infty }\frac{\omega \prime \varepsilon_{2}\left(\omega \prime \right)}{\left(\omega \prime^{2}-\omega^{2}\right)}d\left(\omega \prime \right)$$

The Kramers-Kroning equation can be used to find the imaginary part, by extracting it from the real part, as follows:

(10) $$\varepsilon_{2}\left(\omega \right)=\frac{8}{2\pi \omega }\sum_{nn\prime }\int {\left|P_{nn\prime }\left(k\right)\right|}^{2}\frac{dS_{k}}{\nabla \omega_{nn\prime }\left(k\right)}$$

**Figure 5:**
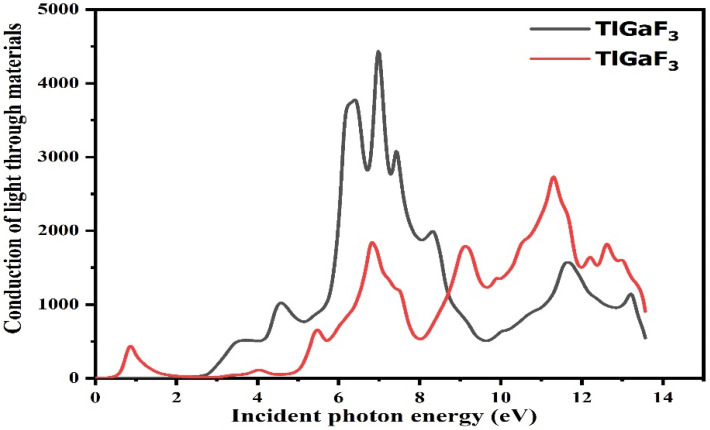
Representation of the relation between the incident light and material response of the given compounds TlMF_3_ (M = Au, Ga).

For optical reactions, the energy range of the photon ranges from 0 to 14 eV. While light absorption is demonstrated from the fictitious portion of the composite’s surface, the dispersive effects are confirmed from the unaffected portion of the surface [28]. The light moves and immersion inside the energy bands is discovered from the fictitious portion by calculating using the dielectric functions, refractive indices, absorption factor, extinction factor, light conductivity, and reflectivity, among other optical properties [29]. The compound TlGaF_3_ rises with the energy between 0 and 14 eV, as shown in Figure 5. The optical conductivity of the compounds TlAuF_3_ and TlGaF_3_ reaches its highest value at 11.1 eV and 7.1 eV, respectively.

### 3.5. Refractive Index

**Figure 6:**
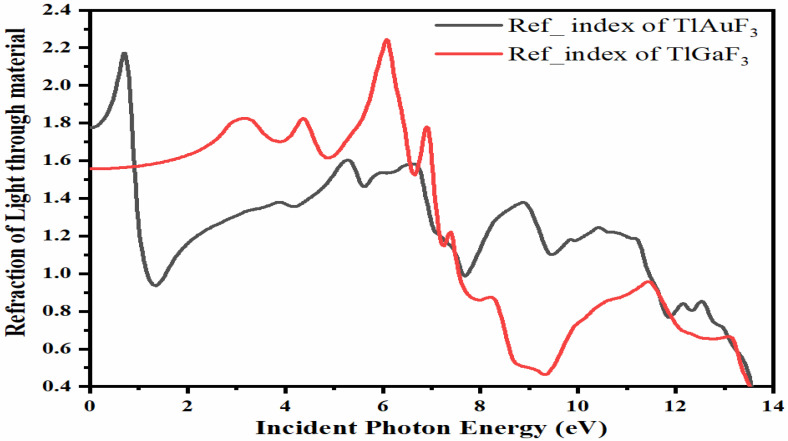
Representation of the relation between refractive indices vs. incident energy for the TMLF_3_ (M= Au, Ga) compounds.

The refractive index of a substance is a dimensionless quantity that indicates how quickly light travels through the substance. Several equations can be used to compute the refractive index [30]. According to Figure 6, the refractive indices, n, of the compounds TlAuF_3_ and TlGaF_3_ exhibit maximum values of 3.25 and 1.6, respectively, peaking at 0 eV energy before progressively declining with several peaks. TlAuF_3_ crested at 0.8, 5.4, 6.5, 8.8, and 10.5 eV, while TlGaF_3_ has peaks at 3.2, 4.4, 6.0, and 7.0 eV.

TlAuF_3_ has its greatest peak, 2.18, of the refractive index at 0.8 eV. Figure 6 demonstrates that the refractive index n(0) at zero photon energy is the maximum, equal to 1.8 for TlAuF_3_ and 1.75 for TlGaF_3_. In contrast, the highest refractive index for TlGaF_3_ is 2.25 at 6.2 eV. Both compounds deviate from the optical signal’s from original direction: for TlAuF_3,_ at less at zero eV, then again at 0.8 eV; and TlGaF_3_ at 6.2 eV.

### 3.6. Absorption Coefficient

Each substance’s absorption factor shows how these substances would react to radiation. According to the absorption coefficient, the frequency significantly affects how incoming photons make electrons and cause them to travel from VB to CB. The aptitude of a substance to engage certain energy photons that strike it is indicated by the absorption coefficient [31]. The absorption constant supports both parts, the real and imaginary components of the dielectric functions. The details are as follows:

(11) $$I\left(\omega \right)=\sqrt{2}\omega {\left[\sqrt{\varepsilon_{1}^{2}\left(\omega \right)+\varepsilon_{2}^{2}\left(\omega \right)}-\varepsilon_{1}^{}\left(\omega \right)\right]}^{1/2}$$

**Figure 7:**
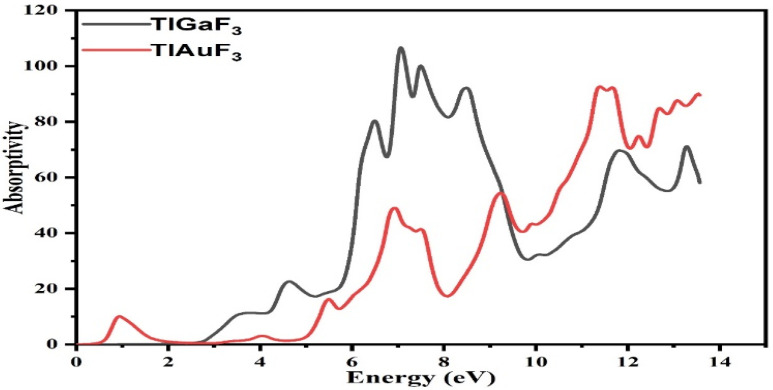
Representation of the absorptivity of fluoro-perovskite TlMF_3_ (M= Au, Ga) vs. response to incident light.

The computed absorption coefficients are shown in Figure 7 for TlAuF_3_ and TlGaF_3_. As seen in Figure 7, whereas the absorption factor for the composite TlGaF_3_ spectral bands starts at 2.8 eV and fluctuates until its peak at 7.0 eV, the absorptivity of the TlAuF_3_ starts at 0.52 eV and reaches a maximum value of 11.1 eV. When radiation intensity reaches a high level, absorption of the radiation occurs, and it reaches the peak. The very sharp cut-off reaction of the materials, which happens largely in this area with such high energy values, explains and proves that these materials can be used in UV optoelectronic devices as an active compound.

### 3.7. Reflectivity

The characteristic surface of a substance is provided when the photons that incident upon it are returned back. The incident light will interact with the composite surfaces a certain amount. Then it will return to its original state once it hits a particular threshold. This is represented by the symbol R(ω) in optical physics. This behavior is determined from the imaginary portion of the dielectric function:

(12) $$\delta \left(\omega \right)=\frac{2w_{ev}\hbar \omega }{E_{O}}$$

**Figure 8:**
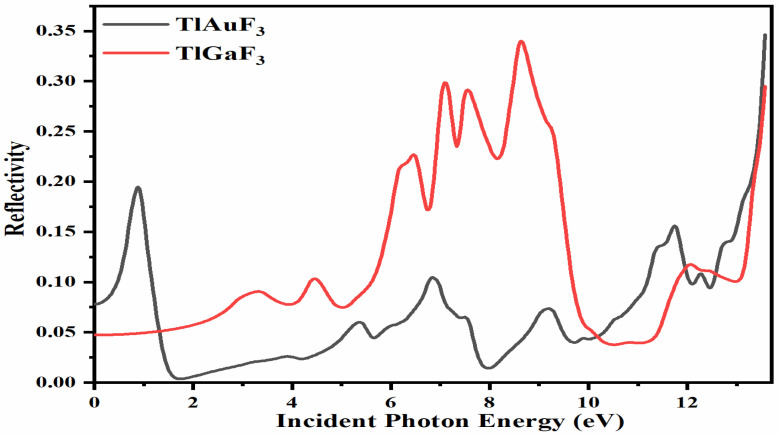
Calculated reflectivity coefficient R(ω) for compounds TlMF_3_ (M = Au, Ga).

Figure 8 demonstrates that at zero photon energy, i.e., 0 eV, the static reflection R(0) was found for the composite TlAuF_3_ to be approximately R(0) = 0.025, while for TlGaF_3_ it was 0.05. Maximum radiation reflection occurs from the compound TlGaF_3_ at the values R(ω)= 0.32, 0.31, 0.33 at 7.0, 7.1, and 11.1 eV. The highest peaks of reflectivity for the compound TlAuF_3_ occur at the values of 0.20 at 0.9 eV and 0.35 at 13.6 eV, as depicted in Figure 8. There is one more crucial point to cover here because it has a connection to the optical part, namely, the fact that these compounds scored poorly here, particularly in the region of the visible and infrared band. This suggests that they do not block light in these spectrums, which is encouraging because researchers use them as anti-reflecting coaters.

## 4. Conclusions

The current study investigated the physical properties of TlMF_3_ (M = Au, Ga) fluoro- is perovskites using the TB-mBJ approach. The lattice parameters at stability for the compound TlGaF_3_ were in the range of 4.5433 and 4.676, whereas those for TlAuF_3_ were in the range of 4.6999 and 4.537. The expected elastic properties are the ECs, anisotropy factor, bulk modulus, Poisson’s ratio, Young’s, and Pugh’s. The Pugh (B/G) ratio demonstrates that the chemicals under investigation are ductile. The values of the computed Poisson ratio further corroborate the ductile nature. Both substances are brittle, stiff, anisotropic, and exhibit ionic bonding. Our research indicates that TlAuF_3_ exhibits indirect band gap behavior at the (X-M) symmetry point, while our estimates indicate that TlGaF_3_ also has an indirect band nature. The estimated results were examined through previously collected experimental and theoretical data and confirmed to be reliable.

## Figures and Tables

**Table 1:** Computed optimized structural properties of TlMF_3_ (M = Au, Ga) from the energy vs. volume fixed by Birch-Murnaghan.

Compounds	ao (Lattice Constant in Å)	B (Bulk Modulus in GPa)	B/(Derivative of Bulk Modulus in GPa)	V0 (Ground State Volume in a.u3)	E0 (Ground State Energy in Ry)
TlAuF_3_	4.6999	44.8590	5.8250	700.5558	−4505.421
TlGaF_3_	4.5433	52.0630	5.8722	632.8047	−79,272.815

## Data Availability

The manuscript included all required data and supplementary information.
